# Cholera Outbreaks in Iran and Duration Time of Outbreaks

**DOI:** 10.4103/0974-777X.52988

**Published:** 2009

**Authors:** Ali Tavana

**Affiliations:** *BMSU, Tehran, Iran.*

**Figure 1:**
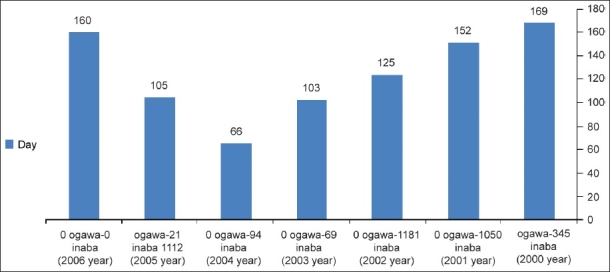
Duration of outbreak by different serogroups during 2000–06

Sir,

This letter focuses on cholera outbreaks in Iran and their duration. *V. cholerae* still causes infection in different countries and no one knows the relationship between cholera outbreaks and their duration in the community. Cholera is still an important infectious disease in the world, particularly in developing countries and could be transmitted primarily by ingestion of *V. cholerae* via contaminated water or food.[1] Cholera is still a major problem in many countries whether in developing or developed countries.[2–7] Little is known regarding the relationship between cholera and duration of the outbreak. However a few studies have showed a strong relationship with other possible links such as rainfall[8], and its seasonal distribution.[9] Also little is known about the duration of cholera outbreaks. The purpose of this study was to discover the relationship between duration and cholera outbreaks. Therefore, in this cross-sectional study the relationship of duration with outbreaks of cholera was investigated for seven years (2000–06) in Iran. In this regard the duration and cases with places of outbreak were recorded across the country. The data were simultaneously input to a computer with the co-operation of the Iran Center of Diseases Control in the Ministry of Health. Data were analyzed and compared with SPSS version 11.5. The results of this study are shown in Table 1 and Figure 1.

**Table 1:** The number of cholera cases in different years (2000–06)

Total	Serogroup			Duration of outbreak	Year
			
	Hikojima	Inaba	Ogawa		
345	0	-	345	169	2000
105	0	1	104	152	2001
118	0	-	118	125	2002
69	0	-	69	103	2003
94	0	-	94	66	2004
1133	0	1112	21	105	2005
*	*	*	*	*	2006

* No cases were reported

The link between climatic factors and bacterial diseases including *V.cholera* has been described before.[10,11] In our study we have found that the duration of outbreak is different year by year, for example in year 2005 in total 1112 cases of cholera in Inaba serogroup and 21 cases of Ogawa serogroup were seen within only 97 days; however in 2001 in total 26 cases of Inaba sero group and 104 cases of Ogawa serogroup of cholera were seen within 151 days. In 2002 118 cases of Ogawa were seen, in 2003 only 69 cases of Ogawa serogroup were seen. In addition in 2004, 94 cases of Ogawa serogroup were reported and finally in 2005, 1133 cases of cholera (1112 cases of Inaba serogroup and 21 cases of Ogawa) were seen. In addition, no cases of Hikojima serogroup were seen at the time of this study. Twenty-four cases of cholera (20 cases of Inaba and 4 cases of Ogawa) were reported in 2006 too within 157 days. It could be concluded that a possible link may be present between duration of outbreak with minimum 66 and maximum 169 days as recorded in our study. In addition, most of the outbreaks happened in warm seasons (end of spring and summer) therefore, the time of outbreak needs much more care during the detection of *V.cholera* by laboratory physicians. Further research could provide more details. It appears that the duration of outbreaks depends on the serogroups. It could be concluded that serogroup variation is present in the outbreaks in the community and perhaps has a link to duration as well.
